# New perspectives on the regulation of germinal center reaction *via* αvβ8- mediated activation of TGFβ

**DOI:** 10.3389/fimmu.2022.942468

**Published:** 2022-08-22

**Authors:** Sébastien This, Helena Paidassi

**Affiliations:** ^1^ Centre International de Recherche en Infectiologie (CIRI), Univ Lyon, Inserm, U1111, Université Claude Bernard Lyon 1, CNRS, UMR5308, ENS de Lyon, Lyon, France; ^2^ Centre de Recherche de l’Hôpital Maisonneuve-Rosemont, Montréal, QC, Canada; ^3^ Département de microbiologie, immunologie et infectiologie, Université de Montréal, Montréal, QC, Canada

**Keywords:** TGFβ activation, alpha(v)-beta8 integrin (αvβ8), IgA B cell response, germinal center (GC) reaction, follicular T helper cells (Tfh), follicular regulatory helper T cell (Tfr), follicular dendritic cell (FDC)

## Abstract

Transforming growth factor-β (TGFβ) is a long-known modulator of immune responses but has seemingly contradictory effects on B cells. Among cytokines, TGFβ has the particularity of being produced and secreted in a latent form and must be activated before it can bind to its receptor and induce signaling. While the concept of controlled delivery of TGFβ signaling *via* α_v_β8 integrin-mediated activation has gained some interest in the field of mucosal immunity, the role of this molecular mechanism in regulating T-dependent B cell responses is just emerging. We review here the role of TGFβ and its activation, in particular by α_v_β8 integrin, in the regulation of mucosal IgA responses and its demonstrated and putative involvement in regulating germinal center (GC) B cell responses. We examine both the direct effect of TGFβ on GC B cells and its ability to modulate the functions of helper cells, namely follicular T cells (Tfh and Tfr) and follicular dendritic cells. Synthetizing recently published works, we reconcile apparently conflicting data and propose an innovative and unified view on the regulation of the GC reaction by TGFβ, highlighting the role of its activation by α_v_β8 integrin.

## Introduction

The humoral arm of adaptive immunity has been at the center of discussion in the recent global COVID-19 pandemic. Efforts to detect and correlate SARS-CoV-2 neutralizing antibodies to protection or to generate these antibodies through vaccination are at the center of the current research landscape (1, 2). More generally, the different processes regulating the B cell responses have long been harnessed for targeted immunotherapies, vaccination being the first and best known. Defects in the regulation of humoral responses are also linked to many human pathologies: B cell hyperplasia, antibody (Ab)-mediated autoimmune disorders, graft rejection, allergy… (3, 4). Understanding the mechanisms underlying the regulation of humoral response reaction is an important goal to identify potential targets to improve vaccine efficacy, develop new therapeutic options for Ab-mediated disorders or propose novel immunotherapeutic strategies.

Pathogen clearance and formation of long-lasting humoral protection through antibody production, requires the activation of B lymphocytes and subsequent differentiation into antibody-secreting plasma cells (PC) and memory B cells (memB). In the vast majority of T-cell dependent (TD) responses, after initial protein antigen (Ag) encounter, antigen-specific B cells interact with antigen-specific T cells and form complex micro-anatomical structures called Germinal Centers (GC).

GC are the main sites where affinity maturation of the antibody response and generation of B cell memory most generally take place. While some B cell responses are GC-independent, the GC reaction (GCR) is key in determining the quality, the amplitude and persistence of the humoral response to TD Ag (5). This complex sequence of events is tightly coordinated by several interdependent cellular and molecular mechanisms which have been extensively described and reviewed over the years (6–10). Among these, cytokine-mediated regulation of the GC, either directly by their action on GC B cells or indirectly to control GC helper cells, is growing in importance.

In this review, we focus on the role of the Transforming Growth Factor β (TGFβ) in the regulation of humoral immunity in the context of T-dependent response. The complex nature of TGFβ biology, and in particular the requirement for its activation, has limited the ability to study this cytokine in the context of the GC regulation. While such concept gained some interest in the field of mucosal immunity, in particular for the regulation of intestinal T cell and IgA responses, the role of α_v_β8-mediated TGFβ activation for regulation of the GCR has been poorly studied. Here, we will highlight the role of TGFβ activation in the regulation of B cell responses, particularly during the GCR.

## TGFβ, a complex pleiotropic cytokine

TGFβ, historically identified as a soluble molecule promoting fibroblast transformation and formation of growth colonies, has since been shown to be a key cytokine for the regulation of the immune system, both for the maintenance of immune homeostasis and the regulation of inflammatory responses (11).

The three isoforms of TGFβ, namely TGFβ1, TGFβ2 and TGFβ3, are produced by a wide diversity of cells, signaling through the TGFβ receptor (TGFβR) on a large range of immune and non-immune cells. In the immune system, it is well accepted that the TGFβ cytokine, TGFβ1 in particular, exerts strong immunomodulatory functions, acts as chemoattractant, promotes immune cell death and, is a critical regulator of immune cell differentiation, particularly of T cells, B cells, Natural Killer T cells and dendritic cells (DC) (12) Overall TGFβ plays a critical role in fine-tuning the immune responses inasmuch as it regulates magnitude and polarization of immune response, contributes to resolution of inflammation, and is critical for immune tolerance (13).

Besides, it is important to note that the studies on the regulation of immune responses by TGFβ have often been limited to the analysis of its secretion and downstream effects. But, among cytokines, TGFβ has a very peculiar position as it is produced and secreted in a latent form and needs to be activated to bind to its receptor and signal (14). Its latent nature is however often overlooked. We will give a brief introduction on the mechanisms of TGFβ activation,

While the role of TGFβ in T cell responses has been extensively studied (15), its implication in the regulation of humoral response has thus far mainly focused on Ig Class A (IgA) Class Switch Recombination (CSR) at mucosal surfaces. The overall function of TGFβ in modulating B cell responses, and in particular during the Germinal Center reaction (GCR), still remains elusive.

In this review, we will first review the instances where regulation of the GCR by TGFβ has been demonstrated, both from the standpoint of its direct effect on B cells and of its ability to modulate the functions of helper cells, namely follicular T cells and follicular dendritic cells (FDC). We will then review what is known of TGFβ activation and IgA CSR as a means to give insight in the direct or indirect regulation of GC by TGFβ-activation. Based on all these studies, we propose several hypotheses for the mechanism(s) by which TGFβ might be activated for the regulation of GC B cell responses.

## Regulation of the GC reaction by TGFβ

### Overall considerations for the TGFβ-mediated regulation of the GC

The GC reaction is a tightly coordinated cascade of events, initiated and driven by the presence of the cognate Ag. The general mechanisms underlying proliferation, affinity maturation and differentiation of B cells in the GC have been largely reviewed elsewhere and will not be described here (5). We highlight here two important parameters of the GCR that relate to its regulation by TGFβ and its activation.

1/The regulation of the GC involves interaction of B cells with multiple cell partners at different steps of the GCR; follicular T cells (namely follicular helper T cells (Tfh) and follicular regulatory T cells (Tfr)) and stromal cells, such as Follicular Dendritic Cells (FDC) (6–10). These cell/cell interactions occur *via* delivery of membrane-bound receptor/ligand signals (thus requiring physical contact and to some extent immune synapse formation) as well as *via* soluble factors. It is now well accepted that the type and stability of the synapses formed between B cells and other cell players of the GC are critical in determining the output of the GC (16). In that particular context, and given the mechanisms for TGFβ activation described below, we believe that controlled delivery of active TGFβ in the context of different combinations of cytokines and/or co-stimulatory factors delivered at cell/cell contact would account for the apparent pleiotropic effect of TGFβ on GC B cell responses.

2/The process of CSR, allowing B cells to switch from IgM and IgD expression to downstream isotypes (such as IgG, IgA, IgE), has long been thought to be an integral part of GC. However, Rocco and colleagues recently extended early observation by Toellner *et al.* to establish that CSR occurs infrequently within the GC (17, 18) and is instead initiated prior to entry into the B cell follicle at the T cell/B cell border (18). This review will detail to some extent the importance of TGFβ in IgA CSR in the mucosal compartment. This subject will be treated separately from the GCR, but we believe that similar mechanisms regulate IgA CSR and the GC reaction *via* activation of TGFβ.

### Direct TGFβ stimulation of B cell influences the GC output

TGFβ is a long-known modulator of antibody responses (19) (**Table 1**). Early *in vitro* studies in the 1980s and 1990s have highlighted the importance of TGFβ signaling for the regulation of B cell responses both in human and mouse. TGFβ was shown to inhibit B cell proliferation *via* growth arrest, to regulate B cell survival *via* induction of apoptosis, to inhibit Ig synthesis and to suppress CSR towards most Ig classes in favor of promoting the IgA class [reviewed in (12, 19, 49)]. In 2000, most of these observations were confirmed *in vivo* by Cazac and Roes (24). Using conditional deletion of TGFβRII on B cells (*Cd19* ^Cre^.*Tgfbr2* ^flox/flox^), they showed that deficient TGFβ signaling in B cells led to reduced lifespan of B2 B cells, expansion of B1-peritoneal B cells, elevated IgG3 responses to a weakly immunogenic antigen, generation of anti-dsDNA antibodies and overall B cell hyperresponsiveness but did not lead to overt clinical autoimmunity. B cell specific TGFβRII deficiency was also associated with a complete loss of IgA. Following these preliminary observations, many studies have further investigated the role of TGFβ for the regulation of mucosal B cell responses, and TGFβ is now widely considered as the master regulator of IgA CSR [reviewed in (29, 30)] (**Table 1**). Conceivably because of this, the study of TGFβ-mediated regulation of B cell responses has often been restricted to its ability to promote IgA CSR and to inhibit B cell proliferation.

**Table 1:** Pleiotropic functions of TGFβ in the regulation of humoral responses.

		Immunostimulating functions of TGFβ		Immunosuppressive functions of TGFβ	
		Effects of TGFβ	Reference	Effects of TGFβ	Reference
**Direct effect of TGFβ on B cells**				*In vitro*, TGFβ limits B cell proliferation by inducing growth arrest	(20–23) (reviewed in (19)
		TGFβ is required for B2B cell survival	(24)	*In vitro*, TGFβ decreases B cell survival by inducing apoptosis	(25–28) (reviewed in (19)
		TGFβ induces IgA CSR	(24) (reviewed in [(29, 30)]	TGFβ controls IgG3 responses, limitsexpansion of peritoneal B1-B cells, limits B cell responsiveness, limits GC in PP	(24)
		TGFβ increases Ab affinity during GC by promoting LZ/DZ transition	(31)	*In vitro*, TGFβ suppresses CSR toward Ig in favor of IgA	(32, 33)
				TGFβ inhibits IgG synthesis	(22)
**Indirect effects of TGFβ on GC via**	**Tfh**	TGFβ is required for Tfh induction in viral influenza infection	(34)	TGFβ limits Tfh frequency and suppresses Tfh function (*in vitro* and *in vivo*)	(35–38)
		TGFβ is required for human Tfhdifferentiation	(39, 39, 40)	*In vitro*, TGFβ inhibits mouse Tfhdifferentiation	(39, 41, 42)
				TGFβ induces Foxp3 expression by Tfh*in vitro*	(43)
	**Tfr**			TGFβ limits auto-immunity by promoting Tfr development	(32, 36, 44)
				TGFβ limits conversion of Tfr into Tfh	(45)
	**FDC**	*In vitro*, TGFβ prevents TNFα-induced apoptotsis of FDC, which may promote a functional FDC network during the GC	(46)		
		TGFβ promotes GC B cell survival by inducing PG production FDC	(47, 48)		

Studies investigating TGFβ signaling specifically on GC B cells reveal that its action might be more refined than initially thought. In agreement with its known inhibitory functions, TGFβ stimulation promotes apoptosis of GC B cells through regulation of Bcl2 family members and inhibition of BCR-mediated rescue from apoptosis (25, 26). Accordingly, the loss of TGFβ signaling in B cell responses induced GC hyperplasia in the PP compartment, chronically stimulated by the gut microflora (24). Interestingly, stimulation of the TGFβRI by BMP7 (member of the TGFβ-superfamily) also promotes apoptosis of human GC B cells through the canonical Smad-signaling pathways (50), showing multiple redundant pathways for controlling GC B cell survival.

More recently, a study analyzing *in situ* pSmad2 signaling in mice by confocal microcopy, revealed that TGFβ signaling in GC B cells promotes the Light Zone (LZ) to Dark Zone (DZ) transition (31). Accordingly, GC B cell specific-TGFβRII deficiency induced accumulation of cells in the LZ, led to decreased apoptosis and in turn decreased antibody affinity. Albright and colleagues thus propose that the specific TGFβ delivery to GC B cells, inside established GC structures, might be driving GC LZ to DZ trafficking rather than IgA CSR (31).

It is important to note that most of these studies did not investigate the source of TGFβ production and delivery to B or GC B cells. Albright and colleagues did however suggest, using conditional deletion of stromal cells, that GC B cell pSmad signaling is at least partially mediated by FDC.

### FoxP3-expressing T cells regulate the humoral response through TGFβ-dependent mechanisms.

FoxP3^+^ T cells have previously been involved in the regulation of multiple aspect of humoral responses through, among other, B cell apoptosis, CSR or Ig production [reviewed in (51, 52)]. Furthermore, several studies demonstrated that FoxP3^+^ T cell-mediated suppression of humoral responses is at least partially mediated by TGFβ (53–56). More recently, it has been demonstrated that CD25^-^Lag3^+^ Treg secreting TGFβ3 suppressed antibody secretion, through mechanisms inhibiting crucial pathways for B cell differentiation and survival (57, 58). In the light of the more recent literature, it is however important to consider that these instances of TGFβ-mediated regulation of B cells by FoxP3^+^ T cells could be mediated by the recently characterized Tfr rather than conventional Treg.

Indeed, 10 years ago, a new subset of Foxp3^+^ regulatory T cells named follicular regulatory T cell (Tfr) has been discovered, that inhibits the GC reaction by directly suppressing Tfh and/or B cells. Since the initial description of Tfr in 2011 (59–61), a growing number of studies has documented the role of these cells in the regulation of GC B cell responses [reviewed in (8, 62, 63)]. While several studies have shown a role for CTLA4 and PD1 in the direct regulation of GC B cells and Tfh, indirect evidence suggest that TGFβ may also be part of the Tfr immunomodulatory machinery. Sayin *et al.* demonstrated that Tfr express elevated levels of the TGFβ-binding protein GARP, at higher levels than conventional Treg (64, 65). McCarron and colleagues also showed that Tfr display significant staining for TGFβ (35). Furthermore, Miles and colleagues, demonstrated that HIV infected tonsil cells displayed a higher frequency of TGFβ expressing Tfr (36), inhibiting B cell responses indirectly through inhibition of Tfh proliferation, ICOS expression and cytokine secretion. More recently, Turner *et al.* showed that the specific deletion of TGFβ1 in all FoxP3-expressing cells (Tfr and conventional Treg) resulted in a high increase of Tfh frequency, suggesting that Tfr-derived TGFβ could regulate Tfh proliferation. This result is in agreement with the study from McCarron and Marie in which they show that TGFβ receptor-deficiency in all T cells leads to the aberrant accumulation of Tfh cells in mice (35, 38). Altogether, these studies point to a critical role of TGFβ in Tfr immunomodulatory functions, even though the precise mechanisms involved in active TGFβ delivery remain to be established.

### TGFβ indirectly regulates GC B cell through regulation of helper cell differentiation and/or function

#### TGFβ a newly established regulator of Tfr development

It is well established that TGFβ is a critical cytokine for the induction of Foxp3^+^ Treg (iTreg) in the periphery and the maintenance of FoxP3 expression by Foxp3^+^ Treg cells (15, 66). A few recent studies also suggest a role for TGFβ in Tfr development. In *ex vivo* models of HIV or HCV infection, important Tfr expansion is detected. Specifically, during HIV infection, this increase in Tfr frequency is only partly mediated by TGFβR signaling, while exosomes secreted by HCV infected hepatocytes, containing large quantities of TGFβ, appear to act directly on T cells to induce Tfr from activated human CD4^+^ T cells (36, 67). Despite some efforts, the authors however failed to fully demonstrate (i) whether this was due to direct TGFβ signaling to T cell or other cells supporting Tfr differentiation (DC, B cells, …) and (ii) whether this was due to *de novo* Tfr differentiation or proliferation of pre-existing Tfr. In parallel, in a mouse model of spontaneous autoimmune development, IL2 and TGFβ synergize *in vivo* to promote Tfr development in the periphery in naïve mice (44). Interestingly, TGFβ stimulation of naïve T cells induces the miR-10a-5p micro-RNA. This micro-RNA, is expressed at high levels on Treg and Tfr and constrains their conversion into Tfh (45). Altogether, these studies suggest that TGFβ signaling on Tfr is important for their induction, their expansion and/or their persistence during the GC reaction.

More recently, Jacobsen and colleagues proposed a mechanism by which Tfh acquire FoxP3 expression and an intermediate CD25^-^ Tfr phenotype in the late stages of the GCR (43). They proposed that these late Foxp3-expressing Tfh participate in the termination of the GCR reaction. TGFβ stimulation of Tfh *in vitro* was capable of inducing FoxP3 expression but the *in vivo* demonstration remains to be made (43).

Overall, it seems that TGFβ, in synergy with other cytokines and signals, is a novel regulator of Tfr differentiation. Some contradicting evidence, such as the study by McCarron and Marie demonstrating that T-cell specific TGFβR deficiency did not lead to a decreased Tfr population (35), however highlights that the mechanism by which TGFβ controls Tfr differentiation has not been fully resolved. It is important to note that Tfr remain a recently-described T cell population and that later studies have demonstrated that Tfr encompass multiple subtypes, derived from either natural thymic Treg, peripherally induced iTreg or Tfh, and generated under different stimulation or immunization scheme and pathological models [summarized in (63)]. It is likely that the cellular origin, localization, and mechanisms of induction of each Tfr subtype might dictate a different requirement for TGFβ.

#### The controversial role of TGFβ in the differentiation of Tfh

In the study of human Tfh differentiation it is now well accepted that TGFβ is a critical factor for the differentiation of naïve T cells into Tfh. TGFβ stimulation induces robust *Bcl6*, *Cxcr5*, *Pdcd1* (coding for PD1), *Icos* and *Il21* expression by peripheral blood CD4^+^ T cells (34, 39, 40).

In comparison, studies in mice show a more controversial role of TGFβ. On one hand, early *in vitro* studies, demonstrated that TGFβ stimulation of naïve murine T cells, inhibits the expression of *Il21*, *Bcl6*, *Icos* and *Cxcr5*, and thus the differentiation of Tfh (39, 41, 42). However, blocking TGFβ signaling *in vivo*, using either anti-TGFβ blocking antibody or the TGFβRII CD4^+^ T cell conditional-dominant negative (DN) mouse model (*dnTGFβRII)*, did not alter the frequency of Tfh in models of NP-KLH immunization and influenza infection (35, 41, 68). Interestingly, while *dnTGFβRII* mice have a similar frequency of Tfh than their WT counterpart upon immunization with NP-KLH, these mice spontaneously develop autoimmune symptoms with an important accumulation of Tfh. The authors propose that, while TGFβ does not appear to control Tfh induction, TGFβ signaling may be required for the regulation of their survival (35).

On the other hand, in the context of acute LCMV infection, Marshall and colleagues revealed an important TGFβ signature on Tfh associated with the strong upregulation of genes generally associated with Treg such as *Nt5e* (CD73), *Folr4* (folate receptor 4), *Foxp3*, and *Ikzf2* (Helios) among other (34). Investigating the chromatin organizer Satb1, several studies have shown that TGFβ stimulation of murine T cells silences *Satb1* expression, which in turn promotes Tfh differentiation (40, 69) Using a TCR transgenic TGFβRII CD4^+^ T cells conditional-KO reveals that *in vivo* generation of Tfh following influenza infection required TGFβ signaling on T cells. In this context, TGFβ inhibits mTOR signaling in T cells and dampens IL2 responsiveness allowing for Tfh differentiation (34). It is important to note that the influenza neuraminidase enzyme can promote the cleavage of latent TGFβ in the lung mucosae (70, 71). While this might not be sufficient to explain the discrepancy in TGFβ requirement for Tfh induction in the study by Marshall *et al*, as compared to the other murine approaches, it is important to note that a wide variety of potentially unknown biases can be at play between these studies. Thus, while TGFβ is clearly required for human Tfh differentiation, it is, to this day, difficult to understand the disparity found in the role of TGFβ for murine Tfh differentiation.

#### Effects of TGFβ on Tfh function

In addition to its controversial role in the differentiation of Tfh, TGFβ also has direct suppressive effects on the function of Tfh, which thus indirectly impacts GC B cell responses. In a recent study using co-cultures of GC-Tfh and GC-B cell isolated from human tonsils, O’Connor and colleagues show that addition of TGFβ is sufficient to inhibit the secretion of IL-21 and sCD40L by Tfh. This is associated with a decreased production of IgG. While a direct of effect of TGFβ on GC B cells cannot be excluded in this co-culture model, this study suggests that TGFβ-mediated suppression of Tfh function would be sufficient to regulate GC B cell responses (37).

This hypothesis is supported by earlier studies both *in vitro* and *in vivo*. As discussed earlier, human tonsil Tfr have the ability *in vitro* to inhibit Tfh proliferation, ICOS expression and cytokine secretion (36). Interestingly, neutralization of TGFβ in co-cultures of Tfh and Tfr restored most of IL-21 production by Tfh, a crucial B cell help cytokine (36). In addition, *in vivo*, mice with a TGFβ deficiency in FoxP3-expressing cells (including Tfr) (*Foxp3^cre^.Tgfb1^flox/flox^*) or with impaired TGFβ signaling in T cells (including Tfh) (*Cd4^cre^.Tgfbr2^flox/flox^*) develop fatal autoimmunity associated with increased frequency of Tfh and GC B cells (35, 38). McCarron and Marie show that accumulation of Tfh wasn’t due to an excessive proliferation of Tfh but rather that Tfh lacking TGFβ signaling are resistant to apoptosis (35). In parallel, O’Connor and colleagues recently identified a new regulatory Innate Lymphoid Cell (ILC) population in the follicles of human tonsils and LN named follicular regulatory ILC (ILCfr), which inhibits the ability of Tfh to secrete IL-21 and sCD40L *in vitro* (37). Interestingly, ILCfr secretes TGFβ upon activation and in co-cultures of human tonsil Tfh, GC B and ILCfr cells, TGFβ neutralization was sufficient to restore IL-21 secretion by Tfh as well as IgG levels by GC B cells (37).

Altogether, these studies demonstrate that TGFβ affects Tfh function (Tfh survival, Tfh expression and secretion of critical B cell help factors) and thus indirectly regulates GC B cell response and autoimmunity. The relative contribution of Tfr and ILCfr in the secretion of TGFβ for the regulation of the ability of GC Tfh to provide B cell help remains to be established.

#### TGFβ-dependent regulation of FDC survival and function

Follicular Dendritic Cells (FDC) form a dense network of stromal cells involved in different aspects of GC B cell proliferation and selection. These cells are critical in the formation of the GC and provide multiple soluble factors instrumental for the correct development of the GCR (9).

Apical LZ FDC have been shown to express the TGFβR (72, 73). Following these early observations, Park *et al.* first described *in vitro* that TGFβ-induced Smad2 signaling in FDC-like cell lines induced a decreased expression of the death receptor signaling pathway: Fas and Caspase 8 (46). TGFβ signaling may thus play a role in preventing Fas-mediated apoptosis of FDC and thus to maintain a functional FDC network throughout the GCR.

Additionally, studies report that TGFβ stimulation of human FDC-like cell promotes, in synergy with IL1β, the production of the Prostaglandin-endoperoxide synthase 2 (Cox-2) enzyme, and in consequence promotes Prostaglandin production (47, 48). Prostaglandin presentation by FDC-like cells *in vitro* actively participates in promoting GC B cell survival (74). Therefore, TGFβ may also indirectly participates in the promotion of the GCR *via* regulation of FDC functions.

Overall, TGFβ appears to have multiple and potentially opposing effects on the GCR. When acting directly on B cells, for example TGFβ has been shown to induce GC B cell apoptosis, and to promote LZ/DZ trafficking and thus increased Ab affinity. Regarding the indirect effects of TGFβ, on one hand TGFβ promotes FDC survival and therefore supports the GC reaction, while on the other hand it promotes Tfr expansion and persistence, which inhibit GC B cell. Finally, its role in regulating Tfh development remains controversial. Altogether these observations highlight that whether acting directly on B cells or indirectly *via* GC helper cells, the role of TGFβ is to this day not entirely resolved and requires further investigation. We believe that these apparent contradictions might be due to the specific nature of TGFβ that needs to be activated to signal.

## α_v_β8-mediated TGFβ activation is key for bioavailability of TGFβ in the immune system

### Latent-TGFβ requires activation prior to signaling

**Figure 1:**
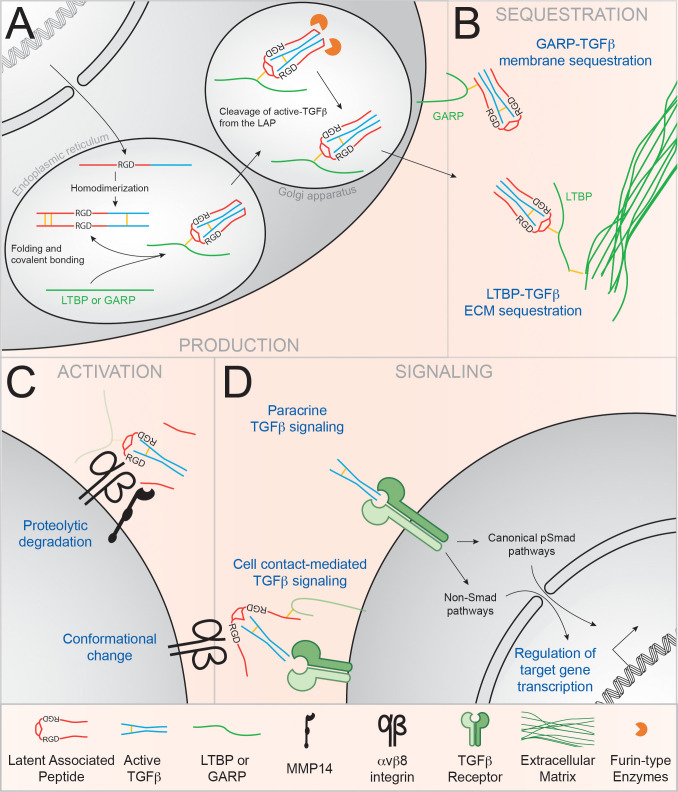
α_v_β8 integrins regulate TGFβ bioavailability in the immune system. TGFβ is produced by cells as an inactive complex and must be activated in order to bind to its receptor and signal. **(A)** Transcription of *TGFβ* produces a homodimeric propeptide containing the active TGFβ molecule (blue) and the Latent Associated Peptide (LAP; in red). In the endoplasmic reticulum, association with the LTBP or GARP ‘chaperone’ proteins (green) enhances proper folding of the latent complex. In the Golgi apparatus, LAP-TGFβ is cleaved by Furin-like enzymes, but active TGFβ stays non-covalently bound to the LAP and forms the Small Latency Complex or latent TGFβ (79) **(B)** Upon secretion, latent TGFβ is sequestered to the ECM through binding of the LTBP or anchored at the plasma membrane by GARP. **(C)** Binding of α_v_ integrins to the RGD tripeptide motif in the LAP induces the dissociation of TGFβ from the LAP *via* the recruitment of metalloproteases such as MMP14 (78). Alternatively, conformational changes can allow TGFβ binding to its receptor without the release of active TGFβ (80). **(D)** TGFβ binding to the TGFβ receptor induces signaling *via* the canonical phospho-Smad (pSmad) pathway, or through the alternative MAPK, Small GTPases and PIP3K pathways.

TGFβ has a very peculiar position among cytokines, as it is produced in tissues in a latent form and must be activated in order to bind to its receptor and enable subsequent signaling and functions. The biology of TGFβ production and activation has been extensively reviewed in several fields of biology (75–78). **Figure 1** recapitulates the mechanism for TGFβ production, sequestration in the tissues, activation and signaling. It is important to note that TGFβ, is overall largely available in tissues and in the serum in a latent form (**Figure 1A**) (81). TGFβ can be found both in the extracellular matrix and bound to cell surface through the recently identified Glycoprotein-A Repetitions Predominant protein (GARP) (**Figure 1B**) (75, 77, 79). Hence, despite some regulation of TGFβ signaling being made at the level of TGFβ secretion by immune cells, most of the regulation of TGFβ bioavailability and downstream signaling is done at the level of its activation.

Many pathways for TGFβ activation have been described (75), however α_v_ integrin-mediated activation of latent TGFβ appears to be one of the most prominent pathway for TGFβ activation *in vivo*. Integrins are heterodimeric transmembrane adhesion molecules, composed of one alpha (α) and one beta (β) subunit, that mediate cell-cell and cell-extracellular matrix interactions (82). α_v_ is the most promiscuous of α subunits, pairing with five different β integrins (β1, β3, β5, β6 and β8). The α_v_ integrins have been implicated in many different cell functions, but their ability to bind and activate latent TGFβ is of particular importance to immune regulation, licensing α_v_-expressing cells to the control of several immune processes (75–77).

Briefly, binding of TGFβ by α_v_ integrins is mediated through an Arginine-Glycine-Aspartate (RGD) tripeptide present at the surface of the LAP of latent TGFβ (**Figure 1C**). Activation of latent TGFβ follows its binding to α_v_ integrins. While many ways of TGFβ activation have been described *in vitro* (proteolytic degradation, deglycosylation, or physicochemical factors (ROS, low pH condition or UV radiation) (75), α_v_ integrin-mediated activation of latent TGFβ appears to be the most important pathway for TGFβ activation *in vivo*. Mutation in the RGD sequence in the TGFβ LAP, which disrupts the α_v_ integrin-binding site (*Tgfb1^RGE/RGE^* mice), indeed recapitulates many of the phenotypes of the TGFβ knockout (KO) mouse (83). Thus, through their involvement in the conversion of latent TGFβ to a form that binds and signals on its receptor, α_v_ integrins have the intriguing ability to regulate TGFβ activation and bioavailability.

While α_v_β1, α_v_β3 and α_v_β5 have been shown to bind and activate latent TGFβ in the context of fibrosis and fibroblast differentiation and function (84–89) its contribution to the regulation of TGFβ-dependent immune responses *in vivo* has not been demonstrated to this day. On the contrary, deletion of the high affinity binding integrins, α_v_β6 or α_v_β8, causes failure of effective TGFβ signaling *in vivo* as both β6- and β8-deficient mice develop inflammation (90, 91). Thus both α_v_β6 and α_v_β8 integrins have been shown to activate TGFβ for the regulation of immune responses (78) (76). However, while β6 KO mice only have a mild phenotype, β8-deficient mice phenocopies mice with a selective loss of α_v_ integrin-mediated TGFβ1 activation (83). This difference in phenotype might be explained by two observations. First, α_v_β8 integrin has a divergent cytoplasmic domain compared to other α_v_ integrins and its constitutionally open conformation likely confers an advantage for the binding of TGFβ without prior inside-out signaling (92–94). Furthermore, α_v_β6 expression seems to be restricted to epithelial cells, contrary to α_v_β8 that has been shown to be expressed by inflammatory fibroblasts and specific immune cell populations (cDC1, Treg). Therefore, because α_v_β8-activation of TGFβ is emerging as critical regulator of TGFβ immune responses, and because α_v_β6 has, to this day, never been associated with the regulation of B cell, only the role of α_v_β8-mediated activation will thus be reviewed here.

Currently two main mechanisms for α_v_β8-mediated TGFβ activation are now being accepted, both involving a third molecular partner [Reviewed in (95)] (**Figure 1C**). On one hand, it has been demonstrated *in vitro* that the Matrix metalloproteinase-14 (MMP14, also called MT1-MMP) is required for the α_v_β8-dependent activation of TGFβ, suggesting a cleavage-dependent local release of active TGFβ (**Figure 1C**) (96). Since then, the importance of MMP14 in the *in vitro* and *in vivo* activation of TGFβ has been demonstrated in multiple context (endothelial function, bone development and pathology, senescence and cancer progression…) (97–102). On the other hand, a few studies have demonstrated the importance of the GARP molecule in “chaperoning” the latent TGFβ molecule for α_v_β8-mediated activation (103–105). The crystal structure of the GARP-TGFβ complex was recently elucidated and showed that LAP binding by GARP allows the further binding of TGFβ by α_v_β8 in a conformation allowing an important flexibility (80, 106). The authors suggest that presentation of latent TGFβ by GARP to α_v_β8 integrin might allow for active TGFβ to be presented to the TGFβR without the need of active soluble TGFβ release at a cell/cell synapse, TGFβ thus remaining membrane bound.

### Regulation of α_v_β8 expression, a checkpoint for the modulation of TGFβ-dependent immune responses

As mentioned earlier, α_v_β8 is a decisive factor for understanding TGFβ-regulated immune responses, especially in the mucosal interfaces. We and others have previously established the key role of α_v_β8 on DC for regulation of intestinal T cell responses *via* the presentation of activated TGFβ to naïve T cells (107–109). Additionally, α_v_β8 expression by Treg themselves is also critical for regulating overt effector T cell-mediated inflammation in the gut (110). Since then, α_v_β8-mediated activation of TGFβ has been involved in the regulation of other TGFβ-dependent immune processes such as IEL generation, regulation of intestinal inflammation, etc. (111–117). This is partly reviewed in (78).

These studies, however revealed two key properties of α_v_β8-mediated TGFβ activation that, we believe, will be important to understand the TGFβ-dependent regulation of the GCR. First α_v_β8-mediated TGFβ activation requires cognate interaction between DC and T cells (116). Hence, reinforcing our assumption that understanding cell/cell contact in the GC is critical. Second, α_v_β8 is expressed in a stable extended-closed conformation, which is not affected by ligand binding or ‘inside-out’ signals, hence excluding the possibility of a contractility-dependent mechanism for α_v_β8-mediated TGFβ activation. The key mechanism for regulating TGFβ activation and thus TGFβ signaling is therefore through the expression of β8 gene (*Itgb8*) (108, 118–121).

In summary, the large availability of TGFβ in tissues and serum, mostly in a latent form (81), and the various pathways for its activation have been powerful arguments to establish that the regulation of TGFβ bioavailability is not only dependent on its secretion but also and most importantly on its activation. In the context of the immune system, α_v_β8-mediated TGFβ activation is emerging as a dominant, if not major pathway for the *in vivo* regulation of activated TGFβ availability and regulation of TGFβ-dependent T cell responses. However, very little is known about the role of α_v_β8-mediated TGFβ activation in regulating B cell responses.

## α_v_β8-mediated activation of TGFβ is required for optimal mucosal IgA responses

**Figure 2:**
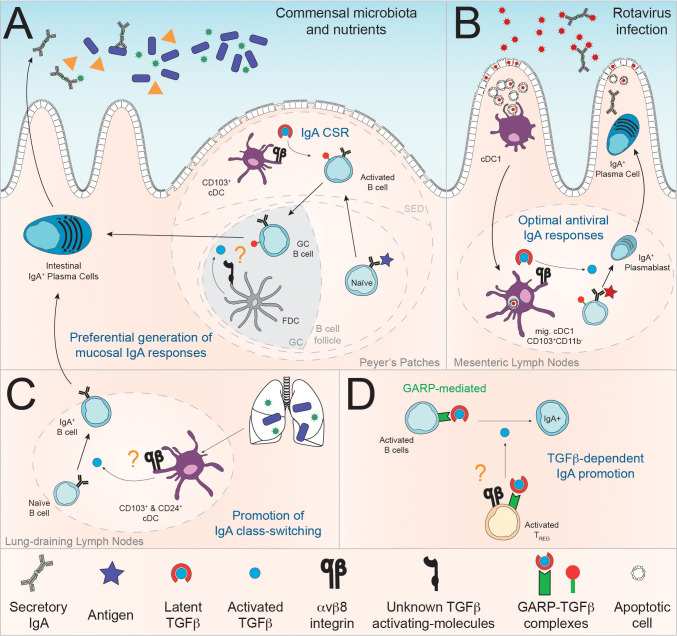
Demonstrated and putative roles of α_v_β8-mediated activation of TGFβ in the regulation of mucosal IgA responses. **(A)** At steady state, Reboldi and colleagues suggest that α_v_β8 expression by Peyer’s Patches CD103^+^ cDC is required for the induction of IgA response to the commensal flora (122). Additionally, TGFβ activation by FDC through mechanisms yet to be determined might also be a mechanism for the promotion of IgA response in the PP (123) **(B)** In the context of intestinal rotavirus infection, MLN cDC1, migrating from the LP, are required for optimal RV-specific IgA response, in part promoted *via* α_v_β8-mediated activation of TGFβ (124). **(C)** In lung-draining LN, the ability of individual cDC subpopulation to induce IgA response to microbiota *via* TGFβ production is correlated with their expression of the *Itgb8* transcript (125). **(D)** Despite lack of formal demonstration, we propose that, given their molecular arsenal and TGFβ-dependent function in the intestinal mucosae, α_v_β8-expressing Foxp3^+^ T cells (Treg) could activate GARP-bound TGFβ on B cells for induction of IgA responses (104, 110). More generally, the expression of GARP-TGFβ complexes by B cells suggest that activated B cells themselves could be a physiologically relevant source of latent TGFβ (126, 127).

TGFβ is the master regulator of IgA class switch recombination but the importance of its activation for this purpose was, until recently, unknown (29, 30). We will focus on the demonstrated and putative mechanisms of α_v_β8-mediated activation of TGFβ by the various B cell partners during IgA response, namely, conventional DC (cDC), T cells (FoxP3-expressing T cells in particular), and FDC (**Figure 2**). It is important to note that despite the high abundance of TGFβ in the IgA PC residency niche, the regulation of PC biology by TGFβ remains to be established. Here, we will thus focus on the well-established role of TGFβ in the induction of IgA responses.

Of note, the origin of latent TGFβ is not precisely investigated but can likely be attributed to mucosal cDC, Tfh and/or FDC, or alternatively non-immune cells such as epithelial cells and fibroblasts, an important sources of latent TGFβ in gut and lung tissues (29, 128–131). Additionally, B cell can also produce TGFβ (131), and this ability has been linked to their cell-intrinsic ability to promote IgA response, placing B cell as a likely important source of TGFβ for IgA responses (132). Furthermore, mature B cells can express the TGFβ-docking GARP protein following B cell receptor and Toll Like Receptor (TLR) stimulation (126, 127), with GARP expression by B cells being important for optimal fecal IgA responses (126, 127). Campbell and colleagues additionally propose that α_v_β8 activation of GARP-bound TGFβ can promote cis-presentation of active-TGFβ on the TGFβ-producing cell (See **Figure 1D**) (80). Altogether, these observations suggest that GARP physically takes TGFβ produced by B cells to the immune synapse and that GARP-bound TGFβ is an important source of TGFβ in the regulation of IgA responses (**Figure 2**).

### α_v_β8-mediated activation of TGFβ by mucosal DC regulates IgA responses

We and others have shown that mucosal cDC, and in particular mesenteric lymph nodes (MLN) migratory cDC, i.e migrating from the intestinal *lamina propria* (LP) to the MLN, specifically express α_v_β8 integrin, which licenses them to activate TGFβ and regulate TGFβ-dependent immune T cell responses (108, 109, 119). More recently, using a newly established reporter mouse model for β8 integrin (*Itgb8)* gene expression, we have shown that *Itgb8* is preferentially expressed by a large proportion of MLN migratory type 1 cDC (cDC1) and to a smaller extent by ~10% of MLN migratory type 2 cDC (cDC2). Additionally, a small fraction (~10%) of Peyer’s Patches (PP) cDC1 also express the β8 integrin subunit (124). Ruane and colleagues also demonstrated that α_v_β8 is expressed, at least at the RNA level, by cDC1 and cDC2 in the lung draining lymph nodes (125). It is interesting to note that α_v_β8 expression by cDC in naïve mice is restricted to mucosal associated lymph nodes as it not expressed in the spleen (119, 125). Consistent with this observation, we have shown that factors, commonly associated with the mucosal compartment such as Retinoic Acid (RA), microbial components, TLR ligands – CpG in particular - and TGFβ itself, promotes *Itgb8* expression on non-mucosal cDC (119, 122). These observations suggest a functional specialization of mucosal cDC for the regulation of mucosal-associated TGFβ-dependent immune responses.

Three studies recently started addressing the role of α_v_β8-mediated activation of TGFβ in the regulation of IgA B cell responses. First, Ruane and colleagues showed in the lung mucosae, that both lung cDC1 and cDC2 promote the generation of protective IgA responses in a TGFβ-dependent manner. This ability correlates with higher expression of the β8 subunit as compared to other antigen presenting subsets, suggesting a role for α_v_β8 expression in promoting lung anti-microbiota IgA responses (125). Second, Reboldi and colleagues have shown *in vitro* using a cDC:B cell coculture model that murine PP cDC from naïve mice are able to promote IgA response in a β8-dependent manner. *In vivo*, they have further demonstrated that conditional KO of *Itgb8* on all CD11c-expressing cells, including cDC (*Cd11c^Cre^ x Itgb8^flox^* mice) or injection of an anti-β8 blocking antibody impairs the formation of steady state IgA^+^ GC B cells (122). Importantly the relative contribution of CD11c-expressing cells (cDC1, cDC2 or other CD11c^+^ cells, including subsets of T cells and ILCs) responsible for α_v_β8-mediated regulation of PP IgA response requires further investigating. Finally, our group has shown that MLN cDC1 but not MLN cDC2 can promote IgA responses *in vitro via* α_v_β8-mediated binding and activation of latent TGFβ. Interestingly, targeted deletion of *Itgb8* in cDC1 (*Xcr1^Cre^ x Itgb8^flox^*) did not significantly alter IgA responses in naïve mice. However, in the context of viral enteric infection, such as the intestinal rotavirus infection, α_v_β8 expression by MLN cDC1 is required for the optimal generation of anti-viral IgA responses (124).

Furthermore, as alluded to earlier, it is thought that the timing of TGFβ delivery and the type of synapse established is an important factor to consider when thinking about TGFβ activation. Interestingly, Reboldi and colleagues propose that the α_v_β8-mediated presentation of activated TGFβ occurs in the sub-epithelial dome of the PP, where prolonged cell/cell conjugates between CD11c^+^ cells and B cells can be observed, prior to the entry of activated B cells into the GC (122). This is consistent with the description that CSR occurs infrequently inside the GC but rather prior to the entry into the B cell follicle (17, 18). While Roco and colleagues show that CSR is initiated at the T cell/B cell border and suggest that the signal for CSR are given by pre-follicular helper T cells, the study by Reboldi and colleagues further suggests that cDC could also provide important and complementary factors for IgA CSR following initial antigen encounter in the mucosal compartment. While we have not investigated the timing of active TGFβ delivery by MLN cDC1 to B cells during rotavirus infection (124), we propose that cDC, migrating from the lamina propria to the MLN, could present activated-TGFβ to B cells concomitantly with the presentation of native antigen coated on their surface, as previously demonstrated (133, 134).

Here it is critical to note that these studies have not formally demonstrated that the α_v_β8-mediated TGFβ activation is required for the induction of IgA responses from naïve B cells *per se* – through the quantification of α-germline transcript or α-circle transcript (CTα) – but rather shows that α_v_β8 is required for the promotion of optimal IgA responses as a whole. Additionally, α_v_β8-mediated TGFβ activation is not the only mechanism by which cDC can support IgA responses; other mechanisms of TGFβ activation might be in place as well as other TGFβ-independent redundant and complementary mechanisms which have already been described (BAFF, APRIL, IL6 and RA secretion) reviewed in (135).

Nevertheless, these studies point to a critical role of α_v_β8-medidated TGFβ activation in the molecular toolkit used by mucosal DC, both cDC1 and cDC2, for the promotion of IgA responses. Besides, it appears that the nature of the initial trigger (i.e viral or bacterial) and the tissue in which the immune response is initiated (i.e draining lymph node, PP or mucosa) will determine the type of cDC subset (i.e cDC1 vs cDC2) that mediates the α_v_β8-mediated control of IgA responses.

### Putative role for T cells and FDC in α_v_β8-mediated control of IgA responses

Despite their conventional immunomodulatory function, intestinal FoxP3-expressing Treg are an important regulator of IgA responses. In 2009, Cong and colleagues first formally demonstrated the importance of CD4^+^CD25^+^ Treg in the generation of IgA responses to flagellin in a TGFβ-dependent manner (136). Further studies confirmed that FoxP3^+^ T cells and thymus derived Treg promote the generation of robust and diverse IgA responses in the gut in a TGFβ-dependent manner (137, 138). Kawamoto and colleagues, further propose that FoxP3^+^ T cells differentiate into Tfr to control IgA production in the intestine (139, 140). Intestinal Treg, as well as Tfr, have been shown to express the α_v_β8 integrin (51, 110), the expression of which licenses Treg to activate GARP-bound latent TGFβ (104, 110). While α_v_β8 expressed by FoxP3-expressing T cells is dispensable for maintenance of intestinal immune homeostasis in naïve mice, it was shown to be required for suppression of T-cell-mediated intestinal inflammation. While no data is currently available, the expression of α_v_β8 by Treg and/or Tfr and their ability to control TGFβ-dependent responses, could be one of the mechanisms licensing them to modulate IgA responses and needs further investigation.

In addition to T cell, B cells also interact with stromal cells, and especially FDC which are known to secrete TGFβ (72, 73, 123, 141). In PP, Suzuki and colleagues have shown that FDC-M1^+^ cells (which includes FDC and contaminating MFG-E8^+^ macrophages) express at high level molecules associated with TGFβ activation (α_v_ integrin subunit, Matrix Metalloproteases, CD36) (123). The authors further show that these TGFβ-activating molecules can be robustly induced in PLN FDC after stimulation with mucosal associated factors such as Retinoic Acid (RA) and TLR ligands. Like PP FDC-M1^+^ cells, these “mucosa-imprinted” FDC then display an increased ability to promote IgA responses *in vitro*. In addition, stimulation with RA and TLR ligands reduces the level of LAP-TGFβ1 present at the surface of PLN FDC, suggesting that active TGFβ is cleaved and shed from the FDC surface. Finally, PP FDC isolated from *Myd88-/-* mice or from mice fed with a vitamin A-deficient diet display increased LAP-TGFβ1 present at the cell surface and these mice display markedly reduced intestinal IgA^+^ populations. Altogether these results suggest that FDC are licensed to promote IgA responses through TGFβ activation, most likely in a α_v_β8-dependent manner.

As CSR infrequently occurs in the GC (17, 18), it seems likely that FDC may play a role for induction of IgA CSR in primary follicles during the initial activation of B cells. It is important to note that the studies discussed here don’t directly show whether TGFβ secretion, activation and/or presentation to B cells by FDC is required for the proper induction of IgA CSR. It is also possible that sustained TGFβ signaling by FDC from the initiation (pre-GC) to the termination of the GCR is required for induction of optimal IgA responses. The precise role of FDC-mediated TGFβ activation in the promotion of IgA responses therefore requires further investigation. The role of FDC-mediated activation of TGFβ for regulation of the GCR, outside of induction of IgA CSR, will be discussed in the next chapter.

To summarize, while the role of α_v_β8-mediated activation of TGFβ by DC for IgA responses is now well established, several studies suggest that follicular T cells and FDC could also activate and present TGFβ to B cells for the promotion of IgA CSR (**Figure 2**).

## Fine regulation of the GCR by TGFβ activation

As described earlier (**Table 1**), TGFβ has seemingly contradictory effects on B cell responses during the GC reaction. This reminds of the multiple effects of TGFβ on T cell responses in the gut, where induction of regulatory or inflammatory Th17 cell is dependent on the context of the synapse where TGFβ is presented to naïve T cells. Thus, the apparent multiple roles of TGFβ in the regulation of the GC are likely dependent on the synapse in which TGFβ is presented and delivered to the target cells.

**Figure 3:**
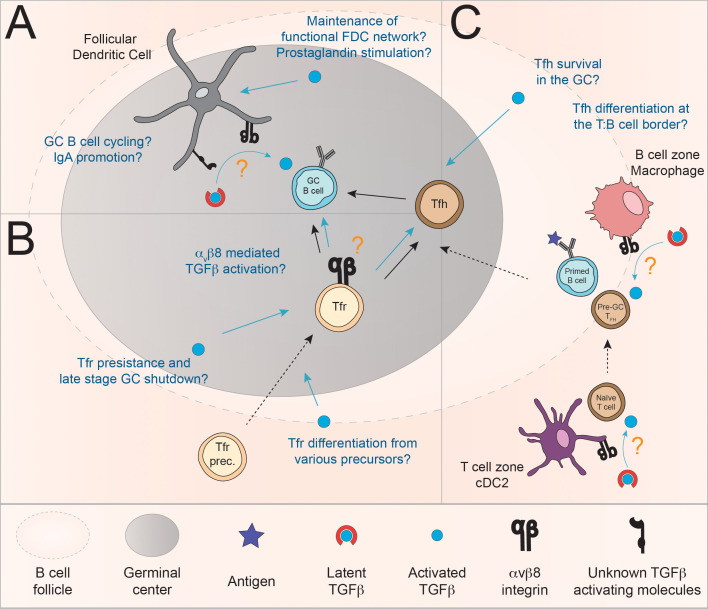
Model for the regulation of the GC reaction *via* the controlled delivery of active TGFβ by α_v_β8 integrin. **(A)** TGFβ signaling in FDC may promote their survival and cytokine production and as such indirectly supports the GC reaction. In addition, given their expression of TGFβ activating molecule, mucosal FDC themselves are a likely source of active TGFβ (123). Furthermore, Albright and colleagues provided evidence that TGFβ produced by FDC may be important for LZ to DZ trafficking of GC B cells. The mechanisms involved may include α_v_β8 although formal demonstration is lacking (31). **(B)** Evidence suggests that TGFβ is required, at least in certain situations, for the induction of Foxp3^+^ follicular T cells (Tfr) (36, 44, 67). In addition, Tfr may be able to regulate GC B cell responses *via* α_v_β8-mediated activation of TGFβ (61, 64, 142). **(C)** α_v_β8-mediated activation of TGFβ by follicular myeloid populations has been involved in the induction of Tfh (143). Additionally, a model in which TGFβ promotes the survival of Tfh in the context of immunization have been proposed (35). In these contexts, the source of active TGFβ is still currently unknown.

Similar to induction of mucosal IgA responses, regulation of the GC involves a plethora of interaction between many cell types (B cells, Tfh, Tfr and FDC) which influence different steps of the GC reaction. Accordingly, regulation of their differentiation, activation, function and/or survival, is critical for an optimal GCR. Here we question the involvement of TGFβ activation, in particular *via* α_v_β8 integrin, in the fine-tuning of GC B cell responses *via* regulation of both the differentiation and the function of these follicular cell populations (Tfh, Tfr and FDC) (**Figure 3**).

### TGFβ-mediated regulation of the GCR by FDC *via* TGFβ activation

The relationship between TGFβ and FDC is complex, as TGFβ was proposed to be a critical regulator of FDC survival and function *in vitro* as well as TGFβ being part of the molecular toolkit of FDC in their promotion of the GC and IgA CSR (31, 46, 47, 123). The question remains as to the mechanism of TGFβ activation for the regulation of GC B cells and FDC biology.

As stated earlier, mucosal associated factors (RA and TLR ligands) have been shown to induce expression of TGFβ-activating molecules in FDC-M1^+^ cells (123). Importantly, FDC are of mesenchymal origin; other mesenchymal cell, such as fibroblasts, have also been shown to activate TGFβ through α_v_- and more specifically α_v_β8-specific mechanisms in the context of lung and liver fibrosis (144, 145). So even though the formal demonstration that FDC can indeed activate TGFβ and present it to B cells remains to be made, these results suggest that FDC may be able to provide active TGFβ for regulation of the GC reaction (123).

While the role of TGFβ in promoting FDC survival and function needs to be confirmed *in vivo*, it is also important to note that the source of latent TGFβ and its mode of activation is not known to this day. Although further investigations are warranted, we speculate that FDC might be able to activate TGFβ in an autocrine manner to promote their survival, at least in the context of mucosal GC. Alternatively, intestinal stromal cell have also been shown to express *Itgb8* and could participate in the paracrine regulation of the FDC network (146). More generally, outside of mucosal associated immune response, the question of α_v_β8-mediated regulation of FDC network remains entirely open.

Overall, the relationship between TGFβ and its activation and FDC populations is two-fold. First, FDC are a likely source of TGFβ production and activation for presentation to B cells during the GCR and second, TGFβ is an important factor in the regulation of FDC survival and cytokine production, thus promoting indirectly the GCR, potentially through the regulation of FDC efficacy but also FDC expansion and contraction throughout the GCR.

### Putative function of α_v_β8 expression by follicular regulatory T cells for TGFβ-mediated regulation of GC B cell responses

Foxp3^+^ Treg and Tfr regulate the GC, at least in part *via* TGFβ (36, 53–56, 64, 65). Furthermore, several clues point towards a role for α_v_β8-mediated TGFβ activation in Tfr function. First, analysis of the differentially expressed genes between Tfr and naïve or effector T cells (microarray) reveals a preferential expression of *Itgb8* on Tfr as compared to naïve and effector T cells (61). Next, in 2017, investigating the transcriptional activity of different regulatory T cells population in a peptide antigens model immunization, it was shown that *Itgb8* is expressed on Tfr and that its expression is controlled through the mTORC1 pathway (64, 142). It is interesting to note that, Jacobsen *et al.* recently proposed that the late-stage contraction of the GC is partially mediated by FoxP3-expressing GC T cells. These cells, in part differentiated from Tfh, display a phenotype closely resembling CD25^-^ Tfr (43).

Altogether, these studies suggest that Tfr have the potential to produce, bind and activate cell-bound TGFβ complexes to control GC B cell responses, potentially in relation with the GC contraction. However, formal demonstration is still pending that α_v_β8 expression by Tfr is required for their immunoregulatory function is required. Similarly, determining which cells (GC B cells and/or Tfh) are directly targeted by Tfr-activated TGFβ during the GCR remains an open question.

In addition, TGFβ is, at least partially, required for the development of some Tfr (**Table 1**). Unfortunately, to this day, there has been no investigation into the mechanisms by which TGFβ is activated and presented to T cell in the context of Tfr differentiation. Given the importance of these cells in regulating the GCR, especially in the context of autoimmune reactions, a better characterization of the TGFβ-dependent mechanisms for Tfr differentiation is highly warranted.

### TGFβ activation in the context of Tfh differentiation

Given the critical role of myeloid cell populations for the activation and presentation of TGFβ to T cells in the context of induced Treg generation in the mucosal environment (108, 109, 119), a couple of studies have investigated the potential role for cDC and macrophages in presenting TGFβ to naïve T cells at the T:B border.

Looking at human pediatric tonsils, Schmitt and colleagues, showed that CD11c^+^ DC present in the T cell zones near GC were positive for TGFβ immunofluorescent staining and that T cells in the vicinity showed robust pSmad staining, indicating active TGFβR signaling (39). In 2019, it was further demonstrated that human tonsil cDC2 and CD14^+^ macrophages are potent inducers of Tfh cells *in vitro*. They further demonstrate that these cells preferentially express OX40L and secrete TGFβ which is required for CXCL13 production by Tfh. Furthermore, both these subsets express the β8 integrin subunit *ex vivo* and after TLR stimulation (143). These data suggest that TGFβ activation by cDC2 and macrophages could potentially regulate Tfh differentiation and/or activation in the human GC environment. Given that cDC2 are found in the T cell zone while CD14^+^ macrophages are rather found in the B cell follicle, the latter colocalizing with Tfh, the authors suggest that these two subsets have a complementary and sequential role in Tfh induction. cDC2 could be responsible for priming pre-Tfh in the T cell zone while CD14^+^ macrophages might instruct the maturation or survival of Tfh in the B cell follicle.

The controversial requirement of TGFβ for Tfh induction and the demonstration that myeloid cells might promote Tfh induction through TGFβ activation warrants further investigation. It is enticing to consider that TGFβ requirement for mouse (and potentially human) Tfh development might be different depending on the type of immunogen and associated danger signals being presented to T cells and that the context (tissue localization, time, co-stimulation, environmental context, etc.) in which TGFβ is activated and presented to naïve T cells, pre-Tfh and mature Tfh might be key to resolve these seemingly contradictory roles of TGFβ on follicular T cell biology.

## Conclusion

From the current literature, it is clear that there is more to TGFβ-mediated control of B cells than just simply IgA CSR and inhibition of B cell proliferation, as TGFβ also regulates Ig CSR decision, Ig production, Ag responsiveness and, in GC B cells, the LZ to DZ transition. The differentiation and/or survival of follicular cell populations (Tfh, Tfr and FDC), which are integral to the GCR, also appear to be regulated by TGFβ, although some controversy remains. Additionally, accumulating evidence points toward a role for α_v_β8 in licensing these populations, along with cDC, for TGFβ-mediated regulation of the GCR. However, TGFβ activation in the context of the regulation of humoral responses, is severely under-investigated and the complete sequence of events allowing the secretion, activation, and presentation of TGFβ for each of these cell types remains elusive. This search is made particularly challenging by the constant discovery of additional layers of regulation of the GCR by TGFβ. For instance, the very recent demonstration that intratumoral CD8^+^ T cells could recruit Tfh to the tumor microenvironment *via* CXCL13 secretion following TGFβ stimulation (147) or the discovery of novel immune populations, such as follicular regulatory Innate Lymphoid Cells (ILCfr), which appear to inhibit the GCR in a TGFβ-dependent manner (37).

It is critical to consider that the regulation of the humoral response involves the interaction of B cells with many different cell types, each of which finely regulates the different steps of the GC reaction (SHM, CSR, proliferation, differentiation, …). Similar to what has been described in the gut, where the induction of Treg and/or inflammatory Th17 cells is dependent on the context encoded at the T cell/DC synapse, the regulation of humoral responses by TGFβ might be dependent on the context in which TGFβ is activated and presented to B cells or to cells regulating B cell responses (FDC, Tfh, Tfr) (78, 148). Because of the production of TGFβ in a latent form, several important parameters must be taken into account to fully understand the importance of TGFβ in regulating the GC: 1/the conditions of production and sequestration of latent TGFβ in the extracellular matrix or at the cell surface; 2/the cells involved and the mechanism by which latent TGFβ is activated; 3/the necessity of TGFβ activation and presentation through cell/cell contact or paracrine secretion, which implies to consider the context of TGFβ delivery, i.e. in conjunction with the presentation of other membrane-bound or soluble factor; 4/the time of delivery, both in the context of individual cell activation and differentiation (e.g. on naïve CD4 T cells, pre-Tfh or fully differentiated Tfh) and in the context of the whole GC (i.e. during initiation, the effector phase or the contraction of the GC)) and 5/which tissue (lymphoid organs vs tissues, mucosal vs peripheral compartment,…) and inflammatory context (steady state vs infection, bacterial vs viral vs fungal vs parasitic infection,…) in which TGFβ is delivered. We believe that only when taking all these considerations of location, timing of cellular interaction and mechanisms of TGFβ activation into account that we will be able to reconcile and resolve the ambiguities of the multiple roles of TGFβ in the regulation of humoral responses in particular in the context of the GCR.

This field of research is rapidly expanding; further studies will be critical in establishing the precise mechanisms of TGFβ-mediated regulation of the GCR, which will surely inform new avenues of treatment for autoimmunity, graft rejection, allergy … Interestingly, animal studies have previously demonstrated that α_v_β8 targeting is possible to treat inflammatory disorders such as encephalitis or lung inflammation and to overcome tumor immune evasion (92, 116, 149, 150). Altogether, these studies suggest that targeting α_v_β8-mediated TGFβ activation could represent a valid strategy to develop immunotherapies for human inflammatory pathologies and cancer (151). Whether this could be extended to humoral responses and could open new therapeutic interventions to re-establish tolerance in antibody-mediated disorders (autoimmunity, allergy, graft rejection) or on the contrary to boost and/or optimize humoral immune responses (in the context of immune-therapy or vaccination) is currently unknown and remains to be investigated.

It is important to note that both B and T cell physiological developments are dependent on TGFβ (19, 152). While it is not discussed in this review, we cannot exclude that some of the experimental setups and evidence described here involve, at least partly, mechanisms linked to development of B or T cell progenitors.

## Author contributions

ST et HP wrote the manuscript. ST designed the figures. HP supervised the study. All authors contributed to the article and approved the submitted version.

## Funding

HP is supported by the Agence Nationale de la Recherche (ANR-20-CE15-0015). ST was supported by a PhD fellowship from the French Ministry of Higher Education and the Cole Foundation Postdoctoral Fellowship.

## Acknowledgments

We would like to thank Dr Thierry Defrance and Prof Olivier Thaunat for many fruitful discussions and critical reading of themanuscript. Servier Medical Art illustrations (under Creative Commons Attribution 3.0 Unported License) were used and modified to make some of the figures in this article (https://smart.servier.com/).

## Conflict of interest

The authors declare that the research was conducted in the absence of any commercial or financial relationships that could be construed as a potential conflict of interest.

## Publisher’s note

All claims expressed in this article are solely those of the authors and do not necessarily represent those of their affiliated organizations, or those of the publisher, the editors and the reviewers. Any product that may be evaluated in this article, or claim that may be made by its manufacturer, is not guaranteed or endorsed by the publisher.

## Abbreviations

TGFβ, Transforming growth factor-β; LTBP, Latent TGFβ binding protein; GARP, Glycoprotein-A repetitions predominant protein; LAP, Latency-associated peptide; TLR, Toll like receptor; BCR, B cell receptor; RA, Retinoic Acid; Ig, Immunoglobulin; Ag, Antigen; cDC, Conventional dendritic cells; FDC, Follicular dendritic cells; Tfh, Follicular helper T cells; Tfr, Follicular regulatory T cell; Treg, Regulatory T cells; PC, Plasma cells; memB, Memory B cells; MLN Mesenteric lymph nodes; PP, Peyer’s patches; SILP, Small Intestinal *lamina propria*; TD, T-cell dependent [B cell responses]; GCR, Germinal center reaction; GC, Germinal Center; LZ, Light Zone; DZ, Dark Zone; CSR, Class-switch recombination; SHM, Somatic hypermutation; KO, Knock-out.
